# The effect of the MR pulse sequence on the regional corpus callosum morphometry

**DOI:** 10.1186/s13244-019-0821-8

**Published:** 2020-02-07

**Authors:** Fahad H. Alhazmi, Osama M. Abdulaal, Abdulaziz A. Qurashi, Khalid M. Aloufi, Vanessa Sluming

**Affiliations:** 1 Department of Diagnostic Radiology Technology, Faculty of Applied Medical Sciences, Taibah University, Madinah, Saudi Arabia; 2 Institute of Translational Medicine, Faculty of Health and Life Sciences, University of Liverpool, Liverpool, UK

**Keywords:** Image quality, Brain morphometry, Corpus callosum, Modified driven equilibrium Fourier transform, Magnetization prepared rapid gradient-echo

## Abstract

**Background and purposes:**

Brain morphometry is an important assessment technique to assess certain morphological brain features of various brain regions, which can be quantified in vivo by using high-resolution structural magnetic resonance (MR) imaging. This study aims to investigate the effect of different types of pulse sequence on regional corpus callosum (CC) morphometry analysis.

**Materials and methods:**

Twenty-one healthy volunteers were scanned twice on the same 3T MRI scanner (Magnetom Trio, Siemens, Erlangen, Germany) equipped with an 8-channel head coil. Two different MR pulse sequences were applied to acquire high-resolution 3D T1-weighted images: magnetization-prepared rapid gradient-echo (MP-RAGE) and modified driven equilibrium Fourier transform (MDEFT) pulse sequence. Image quality measurements such as SNR, contrast-to-noise ratio, and relative contrast were calculated for each pulse sequence images independently. The values of corpus callosum volume were calculated based on the vertex of reconstructed surfaces. The paired dependent *t* test was applied to compare the means of two matched groups.

**Results:**

Three sub-regional CC, namely anterior, mid-anterior, and posterior, resulted in an estimated volume difference between MDEFT and MP-RAGE pulse sequences. Central and mid-posterior sub-regional CC volume resulted in not significant difference between the two named pulse sequences.

**Conclusion:**

The findings of this study demonstrate that combining data from different pulse sequences in a multisite study could make some variations in the results.

## Key points

- Brain morphometry is an important assessment technique to investigate certain morphological brain features of various brain regions, which can be quantified in vivo by using high-resolution structural magnetic resonance (MR) imaging.
- Different MR field strengths and pulse sequences could possibly cause some variations on brain morphometric measurements.
- Combining data from different pulse sequences in a multisite study could make some variations of the results.
- Strict MR parameter options should be determined carefully by automated brain segmentation software packages in order to avoid any misclassification of voxels that are allocated between surrounding tissue types such as gray matter, white matter, and cerebrospinal fluid.

## Introduction

Brain morphometry is an important assessment of certain morphological brain features such as volume, surface, thickness, and shape of various brain regions such as frontal lobe, corpus callosum, and hippocampus that can be measured in vivo by using high-resolution structural magnetic resonance MR imaging technology. It has been used widely to investigate the effect and causes of certain neurological, neurogenerative, psychological, and psychiatric disorders such as epilepsy [1], cognitive impairments [2], autism [3], and schizophrenia [4]. Morphological changes in brain structures could be related to ageing degeneration [5], some neurological disorders [6], and treatment effects [7].

Corpus callosum (CC) is the primary commissural region of the brain consisting of white matter tracts, which connects the right and left hemispheres of the brain, and allows to integrate and transfer the information between the two halves in order to process essential signals such as sensory, motor, and high-level cognitive. CC atrophy is a possible indicator of region- and cell type–specific neuronal degeneration in Alzheimer’s diseases [8]. The relationship between regional microstructural abnormalities of the CC and physical and cognitive disability was studied in the replacing-remitting multiple sclerosis (RRMS) patients, which concludes that regional CC imaging properties differentially explained disability within RRMS patients revealing strong, distinct patterns of correlation with clinical and cognitive status of patients affected by this specific clinical phenotype [9]. Furthermore, regional CC morphometry could demonstrate that specific CC regions may contribute to the cognitive and dysfunction of multiple sclerosis patients [10].

Different MR field strengths, pulse sequences, and parameters could possibly cause some variations on CC morphometric measurements [11–13]. MR field strengths for clinical purposes can vary between 0.2 and 3 T, whereas for experimental purposes up to 11 T. The effect of field strength on MR image quality has been investigated widely [11, 13–15]. High-field-strength MR imaging (1.5 T and above) is considered to acquire high-quality MR images; however, it does not confer higher accuracy in the diagnosis of multiple sclerosis [16]. Furthermore, a simple change of MR parameters, notably spatial resolution, contrast, and filtering, were found to systematically bias the results of automated brain MRI morphometry [12]. Spatial resolution and modification in contrast resulted in relative estimated volume difference of up to 4.28% in cortical GM and 4.16% in the hippocampus between the same MR pulse sequence type with different parameters (1.0 versus 1.2 mm iso-voxel) [12].

Brain structural morphometry studies require a large sample size, and in order to reduce this effect, it is recommended to optimize the imaging acquisition and analysis protocols [17]. Several methods have been applied to obtain brain morphometry such as manual and automated segmentation [18, 19]. Brain morphometry reproducibility was investigated in multi-center 3T MRI sites, which found that longitudinal analysis yields a consistently improved reproducibility across the various sites relative to the cross-sectional segmentation, reducing the variability by about half in most volumetric estimates and in the entorhinal cortical thickness, while not significantly changing the variability in the rest of cortical structures studied [17].

The aim of this study is to investigate the effect of different types of pulse sequence on regional CC morphometry analysis. This will be justified by comparing the image quality and regional CC volumetric analysis among scans of the same participants who have been scanned at the same field strength of 3 T with different MR pulse sequences (magnetization-prepared rapid gradient-echo (MP-RAGE)/modified driven equilibrium Fourier transform (MDEFT)). The main objective of this study was to identify whether scanning at the same MR field strength with different pulse sequences could affect the reliability of brain volumetric analysis.

## Material and methods

### Subjects

Twenty-one subjects (15 males and 6 females) with a mean age of 45 years and a standard deviation (SD) of 12 years participated in this study. All participants received the participants’ information sheet and submitted consent forms prior to the examination. This study has been approved by the National Research Ethics Services (NRES) Committee North West-Liverpool Central.

### MRI data acquisition

**Fig. 1:**
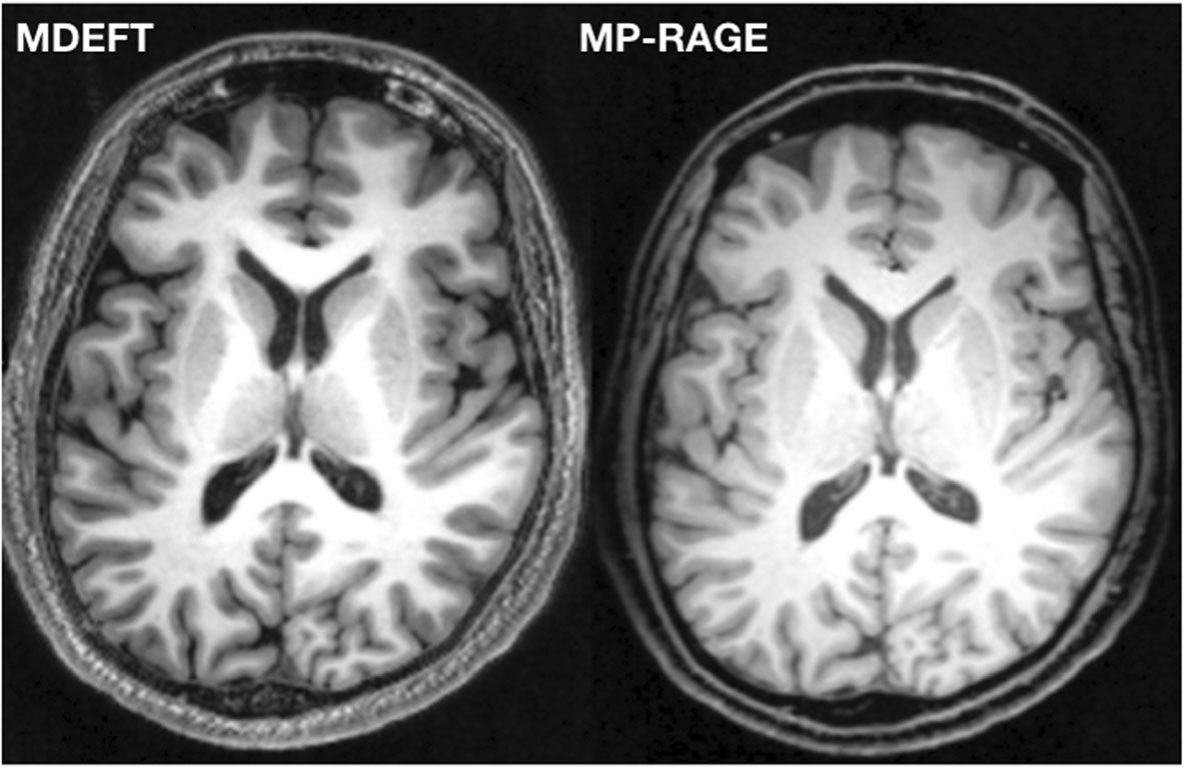
Two different MR pulse sequences were applied to acquire high-resolution 3D T1-weighted images: MP-RAGE and MDEFT pulse sequence

All data were acquired on the same 3T MRI scanner (Magnetom Trio, Siemens, Erlangen, Germany) equipped with an 8-channel head coil. Two different MR pulse sequences were applied to acquire high-resolution 3D T1-weighted images: MP-RAGE and MDEFT pulse sequence (Fig. 1). Scanning parameters for each MR pulse sequence are listed in Table 1. As the acquisition time is quite similar between the two pulse sequences, it is important to mention that no additional acceleration techniques have been applied in this study. MR images were visually inspected in order to approve appropriate image quality and to exclude subjects with visible brain abnormalities.

**Table 1:** Scanning parameters for 3D T1-weighted images: MP-RAGE and MDEFT pulse sequence at 3 T

Scanning parameters	MDEFT	MP-RAGE
TR (ms)	7.92	2300
TE (ms)	2.48	4.37
TI (ms)	910	1100
Flip angle (°)	16	8
FOV (mm^2^)	256 × 256	256 × 256
BW (HZ/Px)	190	190
Number of slices	176	176
Slice thickness (mm)	1	1
Pixel spacing (mm)	1	1
Voxel size (mm)	1 × 1 × 1	1 × 1 × 1
Acquisition time (min:sec)	12:51	12:18

### MRI data analysis

#### MR image quality

Image quality measurements such as SNR and contrast-to-noise ratio (CNR) were calculated for each pulse sequence images. 3D MP-RAGE and 3D MDEFT images for each participant were acquired during the same session in each scanner, maintaining their alignment. The anatomic region of interests (ROIs) include two GM structures (cortex and hippocampus) and two WM structures (corpus callosum and internal capsule), plus a ROI in artifact-free background to sample noise. The ROIs were drawn using the software ImageJ (National Institutes of Health, Bethesda, MD, USA). SNR values for GM and WM were calculated for each subject by averaging across the pair of ROIs corresponding to the respective tissue type. The CNR was then calculated for each subject by averaging across subjects for each imaging method.

#### CC Volumetric analysis

**Fig. 2:**
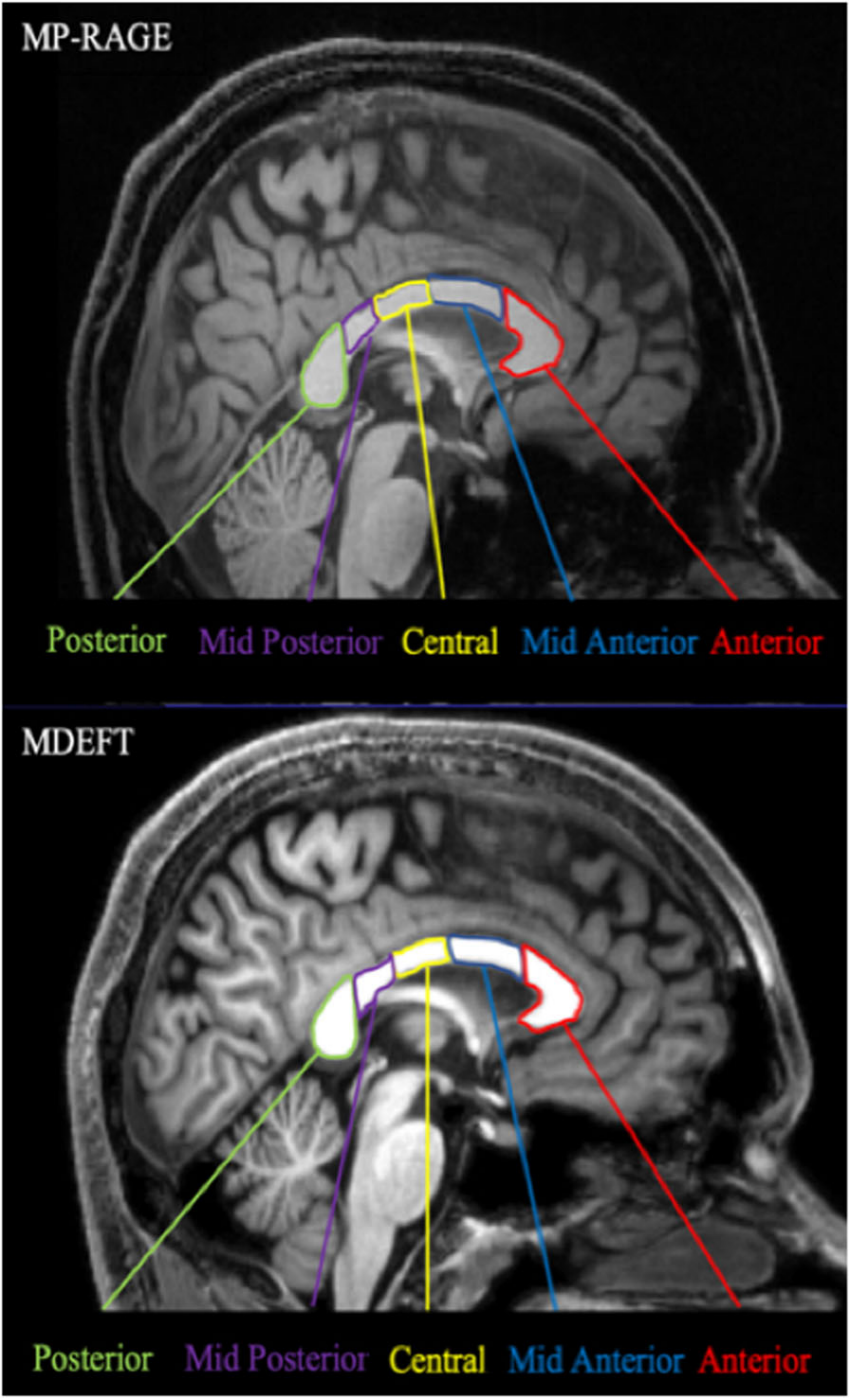
The anatomical locations of CC including five main sub-regions: anterior, mid-anterior, central, mid-posterior, and posterior regions

Surface-based morphometry (SBM) was analyzed using FreeSurfer software version 5.3. It is an automated procedure that derives morphometric measures from geometric models of the cortical surface. 3D T1-weighted images (MP-RAGE and MDEFT) were corrected for intensity bias. Gray and white matter surfaces were reconstructed by segmenting corrected T1-weighted images into GM and WM surfaces. Intracranial volume (ICV) was calculated by adding the values of gray matter, white matter, and cerebral fluid volumes together. The values of corpus callosum volume were calculated based on the vertex of reconstructed surfaces. These data were then smoothed using 10-mm FWHM Gaussian kernel in surface-space in order to improve SNR and inter-subject registration. Based on the anatomical and histological features, CC were segmented into five main sub-regions: anterior, mid-anterior, central, mid-posterior, and posterior regions (Fig. 2).

### Statistical analysis

Normality of residuals was tested using D’Agostino-Pearson normality test in order to quantify how far the distribution is from the Gaussian in terms of asymmetry and shape. Normality test reveals that all data were sampled from a population and follows a Gaussian distribution.

Paired dependent *t* test was applied to compare the means of two matched groups assuming that the distribution of the SNR and CNR data differences follows a Gaussian distribution. Intraclass correlation coefficient (ICC) is applied to perform inter-rater reliability among the MR pulse sequences. The paired dependent *t* test was applied as well as compare the CC volume means of the two matched groups assuming that the distribution of the corpus callosum and intracranial volume data differences follows a Gaussian distribution. All data were analyzed using GraphPad Prism for MacOS version 8.

## Results

### MR image quality

**Fig. 3:**
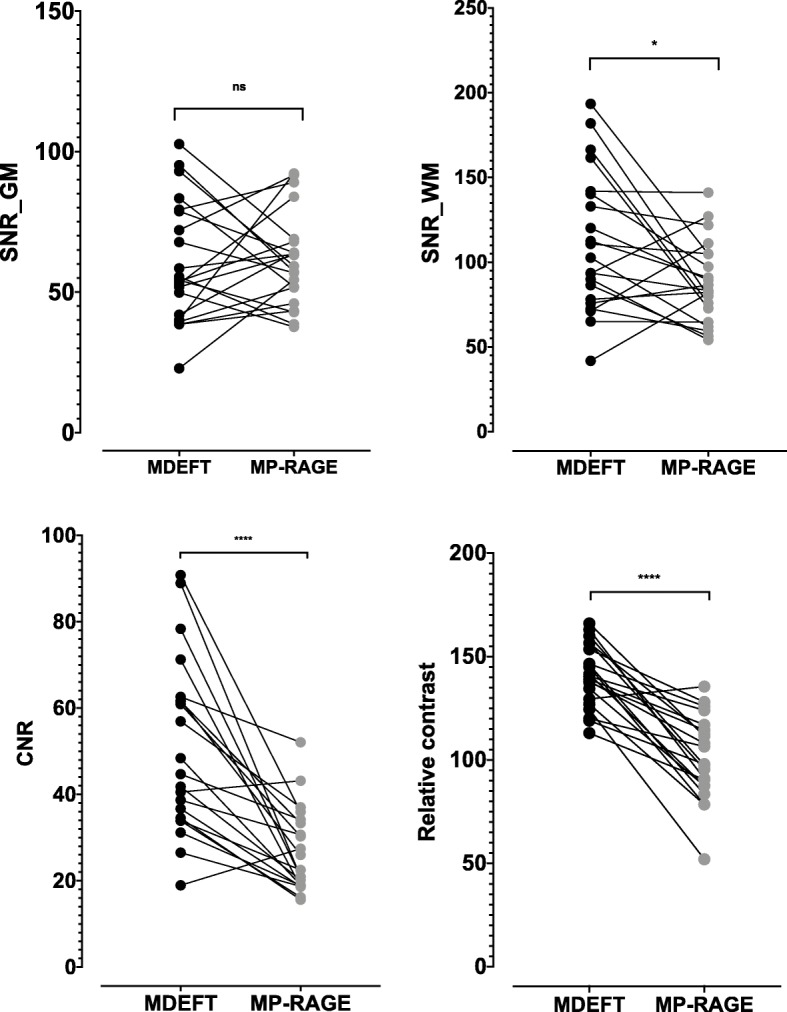
Mean differences graph of SNR in gray matter (GM) and white matter, CNR, and relative contrast between MDEFT and MP-RAGE pulse sequence

No significant differences were found between the SNR values of the gray matter (GM) regions in the MDEFT and MP-RAGE pulse sequences (*t* = 0.15, *p* = 0.87). The mean SNR of the white matter (WM) regions resulted in a significant difference of up to 6% (*t* = 2.52, *p* = 0.02) in the MDEFT pulse sequence (121.77 ± 78.45) compared with MP-RAGE pulse sequence (114.47 ± 73.45). Also, the average CNR and relative contrast resulted in significant difference up to 40% and 30% respectively (*t* = 5.43 and *t* = 8.08 respectively, *p* < 0.0001) in MDEFT pulse sequence (57.94 ± 45.38 and 140.8 ± 15.09 respectively) compared with MP-RAGE pulse sequence (34.69 ± 21.85 and 101.1 ± 20.2 respectively) (Table 2, Fig. 3).

**Table 2:** The mean SNR, CNR, and relative contrast between MDEFT and MP-RAGE pulse sequences

	MDEFT (M ± SD)	MP-RAGE (M ± SD)	Different %	*t* score	df	*p* value
SNR_GM	63.83 ± 33.95	79.78 ± 53.00	20	0.15	20	0.87
SNR_WM	121.77 ± 78.45	114.47 ± 73.45	6	2.52	20	0.02*
CNR	57.94 ± 45.38	34.69 ± 21.85	40	5.43	20	< 0.0001****
Relative contrast	140.8 ± 15.09	101.1 ± 20.2	30	8.08	20	< 0.0001****

*p* values < 0.05 are shown with one asterisk (*), *p* values < 0.01 are shown with two asterisks (**), *p* values < 0.001 are shown with three asterisks (***), and *p* values < 0.0001 are shown with four asterisks (****)

**Fig. 4:**
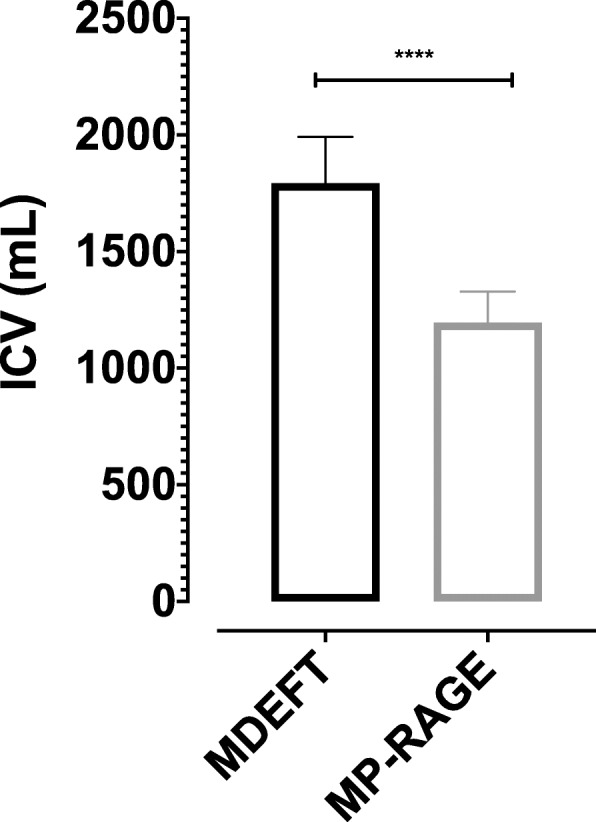
Mean and standard deviation (SD) of intracranial volume (ICV) versus pulse sequences (MDEFT and MP-RAGE). Volumes are in mL

**Fig. 5:**
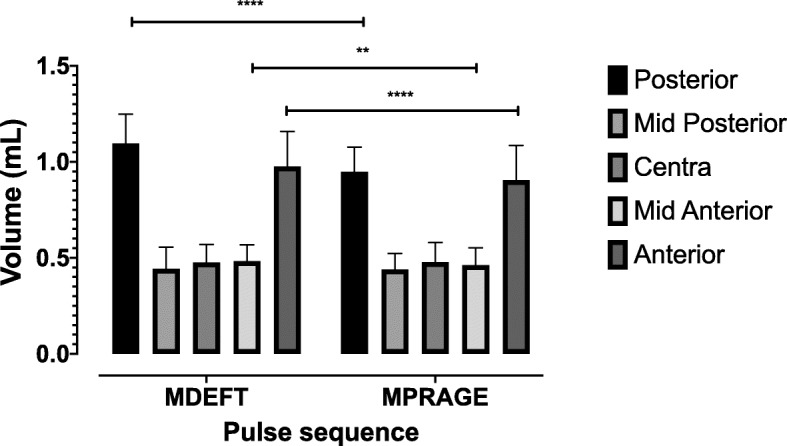
Mean and standard deviation (SD) of volumetric measurements of CC regions versus pulse sequences (MDEFT and MP-RAGE). Volumes are in mL

Total intracranial volume (ICV) was found significantly higher (*p* < 0.0001) in the MDEFT pulse sequence (1792.4 ± 197.8 mL) compared with the MP-RAGE pulse sequence (1195.2 ± 133.1 mL) (Fig. 4). Three sub-regional CC volumes, namely anterior (*p* < 0.0001), mid-anterior (*p* = 0.003), and posterior (*p* < 0.0001) were found significantly larger in MDEFT pulse sequence compared with the MP-RAGE pulse sequence. Central and mid-posterior sub-regional CC volume was found no significant difference between the two pulse sequences (Table 3 and Fig. 5).

**Table 3:** Mean and standard deviation (SD) of intracranial volume (ICV) and volumetric measurements of CC regions versus pulse sequences (MDEFT and MP-RAGE). Volumes are in mL, *ns*, not significant

	MDEFT (M ± SD)	MP-RAGE (M ± SD)	*t* score	df	*p* value	95.10% CI
ICV	1792.4 ± 197.8	1195.2 ± 133.1	13.6	21	< 0.0001^****^	− 689.6 to − 506.6
Posterior	1.095 ± 1.531	0.947 ± 0.129	9.75	21	< 0.0001^****^	0.116 to 0.179
Mid-posterior	0.443 ± 0.112	0.439.7 ± 83.4	0.29	21	0.77 ^ns^	− 0.02 to 0.02
Central	0.476 ± 103.1	0.477 ± 0.103	0.06	21	0.94 ^ns^	− 0.02 to 0.019
Mid-anterior	0.482 ± 0.86	0.462 ± 0.89	3.31	21	0.003 ^**^	0.07 to 0.03
Anterior	0.975 ± 0.182	0.904 ± 0.181	5.80	21	< 0.0001^****^	0.04 to 0.09

*p* values < 0.05 are shown with one asterisk (*), *p* values < 0.01 are shown with two asterisks (**), *p* values < 0.001 are shown with three asterisks (***), and *p* values < 0.0001 are shown with four asterisks (****)

Reproducibility coefficients for volumetric measurements of CC regions in the two pulse sequences (MDEFT/MP-RAGE) are presented in Table 4. Reliability was very good which ranges between 0.89 and 0.95 for all CC regions in the comparison between MDEFT and MP-RAGE pulse sequences. This reliability test resulted in a statistically significant difference (*p* < 0.0001) for all the CC regions between the two examined pulse sequences.

**Table 4:** Intraclass correlation coefficients of callosal morphometric measurements

	Posterior	Mid-posterior	Central	Mid-anterior	Anterior
MDEFT vs MP-RAGE	0.89	0.89	0.90	0.95	0.95

## Discussion

This study examined the reliability of regional CC morphometry analysis between two different pulse sequence (MDEFT and MP-RAGE) at the same MR field strength (3 T) using an automated procedure derives morphometric measures from geometric models of the cortical surface. There is a significant difference in volumes up to 33% between MDEFT and MP-RAGE when evaluating total ICV that might be resulted in the significant differences of CNR and relative contrast up to 40% and 30% respectively. The volumes of three sub-regions of CC (anterior, mid-anterior, and posterior) were found significant differences across the two examined MR pulse sequences. Reliability measurements were found significantly high in all CC regions across the two examined MR pulse sequences.

In this study, we reported that MDEFT pulse sequence acquires a better SNR in WM, CNR, and relative contrast compared with MP-RAGE pulse sequence, while the SNR in GM seems to be similar between these two pulse sequences, which is consistent to similar study by Tardif et al. who reported that MDEFT pulse sequence achieved the highest CNR between WM and GM, and the lowest GM density variability compared with MP-RAGE and FLASH pulse sequences [20]. A more recent introduced sequence MDEFT was suggested to be more favorable than MP-RAGE at high field strengths [21]. It is characterized by a distinct regional bias in GM density due to the effect of transmission field inhomogeneity on image uniformity combined with spatially variant GM T(1) values and the sequence’s T(1) contrast function [20]. Compared with MP-RAGE, MDEFT was proposed to be relatively insensitive to inhomogeneities in the B1 field [22]. SNR, CNR, and relative contrast values obtained from different pulse sequences (MDEFT/MP-RAGE) at the same field strength of 3 T were different. Relying on the findings of the current study and combining and comparing SNR, CNR, and relative contrast data of different pulse sequences (MDEFT/MP-RAGE) at the same field strength of 3 T appears to be possible. Therefore, the results of this study should be carefully assessed when comparing them to results from other field strengths.

Several methods for examining the brain morphometry are relied on high-resolution T1-weighted using (MP-RAGE) or (MDEFT) pulse sequences [11, 23–25]. MP-RAGE offers good contrast between brain tissues: the gray matter (GM), white matter (WM), and cerebrospinal fluid (CSF) [26, 27]. It captures high tissue contrast and provides high spatial resolution with whole-brain coverage [28]. On the other hand, 3D MDEFT sequence has been optimized recently for anatomical brain imaging at 1.5 T and 3.0 T [29]. It is proposed that 3D MDEFT lowers its sensitivity to RF inhomogeneity that is particularly essential at high field strengths where RF focusing effects aggravate B1 inhomogeneity, causing major signal non-uniformity in the MR images [30]. It is worth to mention that bias field correction algorithm is an image preprocessing step usually used for segmentation technique in order to correct low frequency intensity non-uniformity present in MR image data and minimizing segmentation error [31, 32]. It was suggested that the MP-RAGE sequence noticeably improved the outlining of GM and WM and significantly reduced flow artifacts [33]. On the other hand, the MDEFT consists of a saturation pulse and an inversion pulse, which is usually used to reduce radio-frequency inhomogeneity sensitivity and improves brain tissue contrast [30]. MDEFT was suggested to be more satisfactory than MP-RAGE at high field strengths [21].

In this study, the same software pipeline (FreeSurfer) was used to quantify the CC volume in order to eliminate the effect of different software pipelines on brain morphometry measurements. In automated FreeSurfer segmentation, the CC was found appropriate for analysis without manual correction [19]. Furthermore, a constant bandwidth was applied in this study as it represents a potential difference from typical clinical conditions, under which the minimum bandwidth for each unit is generally used [13]. Also, a pilot study had been undertaken in order to assess the segmentation quality of each pulse sequence before starting participant recruitment.

As only healthy subjects were recruited in this study, it is difficult to draw a definite conclusion regarding the effect of different pulse sequences on the diagnostic accuracy for some neurological cases. In addition, automatic segmentation performed for CC morphometry analysis may cause some misclassification of some voxels. In other words, given voxels were allocated in GM and classified as WM or CSF because of the limited resolution of MRI scanner. This limitation was solved by visual inspection and manual correction to ensure that voxels are representing the correct brain tissues. The result of this paper may only apply for the same version of the FreeSurfer pipeline, which needs to be compared with the other versions of the FreeSurfer package in order to evaluate the difference of measured volumes between different FreeSurfer package versions.

## Conclusion

The findings confirm that the MDEFT pulse sequence acquires a better SNR in the WM, CNR and relative contrast compared with the MP-RAGE pulse sequence, while the SNR in the GM seems to be similar between these two pulse sequences. Relying on the findings of the current study, the variation of brain volumetric measurements between different pulse sequences (MDEFT/MP-RAGE) at the same field strength of 3 T seems to be possible. Combining data from different pulse sequences in a multisite study could make some variations of the results. Therefore, the findings of this study should be carefully assessed when comparing them to results from other field strengths and MRI scanner manufactures. Furthermore, strict MR parameter options should be determined carefully by automated brain segmentation software packages in order to avoid any misclassification of voxels that are allocated between surrounding tissue types such as GM, WM, and CSF. Future studies are required to investigate the effect of other factors such as scanner manufacturers, coil channel selections, and other pulse sequences parameters options on the MR image quality and brain morphometry analysis.

## Abbreviations

CC: Corpus callosum
CSF: Cerebrospinal fluid
GM: Gray matter
ICV: Intracranial volume
MDEFT: Modified driven equilibrium Fourier transform
MP-RAGE: Magnetization-prepared rapid gradient-echo
MR: Magnetic resonance
NRES: National Research Ethics Services
RRMS: Replacing-remitting multiple sclerosis
SBM: Surface-based morphometry
T: Tesla
TE: Echo time
TI: Inversion time
TR: Repetition time
WM: White matter

## References

[1] Alhusaini Saud, Kowalczyk Magdalena A., Yasuda Clarissa L., Semmelroch Mira K., Katsurayama Marilise, Zabin Matheus, Zanão Tamires, Nogueira Mateus H., Alvim Marina K.M., Ferraz Victória R., Tsai Meng-Han, Fitzsimons Mary, Lopes-Cendes Iscia, Doherty Colin P., Cavalleri Gianpiero L., Cendes Fernando, Jackson Graeme D., Delanty Norman (2018). Normal cerebral cortical thickness in first-degree relatives of temporal lobe epilepsy patients. Neurology.

[2] Kim H. J., Ye B. S., Yoon C. W., Noh Y., Kim G. H., Cho H., Jeon S., Lee J. M., Kim J.-H., Seong J.-K., Kim C.-H., Choe Y. S., Lee K. H., Kim S. T., Kim J. S., Park S. E., Kim J.-H., Chin J., Cho J., Kim C., Lee J. H., Weiner M. W., Na D. L., Seo S. W. (2014). Cortical thickness and hippocampal shape in pure vascular mild cognitive impairment and dementia of subcortical type. European Journal of Neurology.

[3] Schumann CM, Barnes CC, Lord C, Courchesne E (2009). Amygdala enlargement in toddlers with autism related to severity of social and communication impairments. Biol Psychiatry.

[4] Welch K. A., Stanfield A. C., Moorhead T. W., Haga K., Owens D. C. G., Lawrie S. M., Johnstone E. C. (2009). Amygdala volume in a population with special educational needs at high risk of schizophrenia. Psychological Medicine.

[5] Castellanos F. Xavier (2002). Developmental Trajectories of Brain Volume Abnormalities in Children and Adolescents With Attention-Deficit/Hyperactivity Disorder. JAMA.

[6] Fotenos AF, Snyder AZ, Girton LE, Morris JC, Buckner RL (2005). Normative estimates of cross-sectional and longitudinal brain volume decline in aging and AD. Neurology.

[7] Yang Chung-Yi, Liu Hon-Man, Chen Shan-Kai, Chen Ya-Fang, Lee Chung-Wei, Yeh Lee-Ren (2016). Reproducibility of Brain Morphometry from Short-Term Repeat Clinical MRI Examinations: A Retrospective Study. PLOS ONE.

[8] Hampel Harald, Teipel Stefan J., Alexander Gene E., Horwitz Barry, Teichberg Diane, Schapiro Mark B., Rapoport Stanley I. (1998). Corpus Callosum Atrophy Is a Possible Indicator of Region– and Cell Type–Specific Neuronal Degeneration in Alzheimer Disease. Archives of Neurology.

[9] Caligiuri Maria Eugenia, Barone Stefania, Cherubini Andrea, Augimeri Antonio, Chiriaco Carmelina, Trotta Maria, Granata Alfredo, Filippelli Enrica, Perrotta Paolo, Valentino Paola, Quattrone Aldo (2015). The relationship between regional microstructural abnormalities of the corpus callosum and physical and cognitive disability in relapsing–remitting multiple sclerosis. NeuroImage: Clinical.

[10] Yaldizli Özgür, Penner Iris-Katharina, Frontzek Karl, Naegelin Yvonne, Amann Michael, Papadopoulou Athina, Sprenger Till, Kuhle Jens, Calabrese Pasquale, Radü Ernst Wilhelm, Kappos Ludwig, Gass Achim (2013). The relationship between total and regional corpus callosum atrophy, cognitive impairment and fatigue in multiple sclerosis patients. Multiple Sclerosis Journal.

[11] Abdul-Kareem Ihssan A., Stancak Andrej, Parkes Laura M., Sluming Vanessa (2009). Regional corpus callosum morphometry: Effect of field strength and pulse sequence. Journal of Magnetic Resonance Imaging.

[12] Haller S, Falkovskiy P, Meuli R, Thiran J-P, Krueger G, Lovblad K-O, Kober T, Roche A, Marechal B (2016). Basic MR sequence parameters systematically bias automated brain volume estimation. Neuroradiology.

[13] Maubon Antoine J., Ferru Jean-Michel, Berger Vincent, Soulage Marie Colette, DeGraef Marc, Aubas Pierre, Coupeau Patrice, Dumont Erik, Rouanet Jean-Pierre (1999). Effect of Field Strength on MR Images: Comparison of the Same Subject at 0.5, 1.0, and 1.5 T. RadioGraphics.

[14] Han Xiao, Jovicich Jorge, Salat David, van der Kouwe Andre, Quinn Brian, Czanner Silvester, Busa Evelina, Pacheco Jenni, Albert Marilyn, Killiany Ronald, Maguire Paul, Rosas Diana, Makris Nikos, Dale Anders, Dickerson Bradford, Fischl Bruce (2006). Reliability of MRI-derived measurements of human cerebral cortical thickness: The effects of field strength, scanner upgrade and manufacturer. NeuroImage.

[15] Rutt BK, Lee DH (1996). The impact of field strength on image quality in MRI. J Magn Reson Imaging.

[16] Lee D H, Vellet A D, Eliasziw M, Vidito L, Ebers G C, Rice G P, Hewett L, Dunlavy S (1995). MR imaging field strength: prospective evaluation of the diagnostic accuracy of MR for diagnosis of multiple sclerosis at 0.5 and 1.5 T. Radiology.

[17] Jovicich Jorge, Marizzoni Moira, Sala-Llonch Roser, Bosch Beatriz, Bartrés-Faz David, Arnold Jennifer, Benninghoff Jens, Wiltfang Jens, Roccatagliata Luca, Nobili Flavio, Hensch Tilman, Tränkner Anja, Schönknecht Peter, Leroy Melanie, Lopes Renaud, Bordet Régis, Chanoine Valérie, Ranjeva Jean-Philippe, Didic Mira, Gros-Dagnac Hélène, Payoux Pierre, Zoccatelli Giada, Alessandrini Franco, Beltramello Alberto, Bargalló Núria, Blin Olivier, Frisoni Giovanni B. (2013). Brain morphometry reproducibility in multi-center 3T MRI studies: A comparison of cross-sectional and longitudinal segmentations. NeuroImage.

[18] Despotovic I, Goossens B, Philips W (2015) MRI segmentation of the human brain: challenges, methods, and applications. Comput Math Methods Med 2015:450341 10.1155/2015/450341 PMC4402572 25945121

[19] Guenette Jeffrey P., Stern Robert A., Tripodis Yorghos, Chua Alicia S., Schultz Vivian, Sydnor Valerie J., Somes Nathaniel, Karmacharya Sarina, Lepage Christian, Wrobel Pawel, Alosco Michael L., Martin Brett M., Chaisson Christine E., Coleman Michael J., Lin Alexander P., Pasternak Ofer, Makris Nikos, Shenton Martha E., Koerte Inga K. (2018). Automated versus manual segmentation of brain region volumes in former football players. NeuroImage: Clinical.

[20] Tardif CL, Collins DL, Pike GB (2009). Sensitivity of voxel-based morphometry analysis to choice of imaging protocol at 3 T. Neuroimage.

[21] Ugurbil K, Garwood M, Ellermann J, Hendrich K, Hinke R, Hu X, Kim SG, Menon R, Merkle H, Ogawa S (1993). Imaging at high magnetic fields: initial experiences at 4 T. Magn Reson Q.

[22] Highley JR, Esiri MM, Mcdonald B, Cortina-borja M, Herron BM, Crow TJ (1999). The size and fibre composition of the corpus callosum with respect to gender and schizophrenia: a post-mortem study. Brain.

[23] Abdul-Kareem Ihssan A., Sluming Vanessa (2008). Heschl gyrus and its included primary auditory cortex: Structural MRI studies in healthy and diseased subjects. Journal of Magnetic Resonance Imaging.

[24] Aldhafeeri FM, Alghamdi J, Sluming V, Mackenzie I, Kay T (2012). Neuroanatomical correlates of tinnitus revealed by cortical thickness analysis and diffusion tensor imaging. Neuroradiology.

[25] FIschl B, Dale AM (2000). Measuring the thickness of the human cerebral cortex from magnetic resonance images. Proc Natl Acad Sci U S A.

[26] Mugler JP, Brookeman JR (1990). Three-dimensional magnetization-prepared rapid gradient-echo imaging (3D MP RAGE). Magn Reson Med.

[27] Mugler JP, Brookeman JR (1991). Rapid three-dimensional T1-weighted MR imaging with the MP-RAGE sequence. J Magn Reson Imaging.

[28] Wang Jinghua, He Lili, Zheng Hairong, Lu Zhong-Lin (2014). Optimizing the Magnetization-Prepared Rapid Gradient-Echo (MP-RAGE) Sequence. PLoS ONE.

[29] Deichmann R, Schwarzbauer C, Turner R (2004). Optimisation of the 3D MDEFT sequence for anatomical brain imaging: technical implications at 1.5 and 3 T. Neuroimage.

[30] Thomas David L., De Vita Enrico, Deichmann Ralf, Turner Robert, Ordidge Roger J. (2005). 3D MDEFT imaging of the human brain at 4.7 T with reduced sensitivity to radiofrequency inhomogeneity. Magnetic Resonance in Medicine.

[31] Gispert JD, Reig S, Pascau J, Vaquero JJ, Garcia-barreno P, Desco M (2004). Method for bias field correction of brain T1-weighted magnetic resonance images minimizing segmentation error. Hum Brain Mapp.

[32] Juntu J, Sijbers J, Van Dyck D, Gielen J (2005) Bias Field Correction for MRI Images. In: Kurzyński M, Puchała E, Woźniak M, żołnierek A (eds) Computer Recognition Systems. Advances in Soft Computing, vol 30. Springer, Berlin, Heidelberg

[33] Fellner F, Holl K, Held P, Fellner C, Schmitt R, Bohm-jurkovic H (1996). A T1-weighted rapid three-dimensional gradient-echo technique (MP-RAGE) in preoperative MRI of intracranial tumours. Neuroradiology.

